# The conformation of tetraspanins CD53 and CD81 differentially affects their nanoscale organization and interaction with their partners

**DOI:** 10.1016/j.jbc.2024.107685

**Published:** 2024-08-17

**Authors:** Fabian Schwerdtfeger, Ilse Hoogvliet, Sjoerd van Deventer, Annemiek B. van Spriel

**Affiliations:** Department of Medical BioSciences, Radboud University Medical Center, Nijmegen, The Netherlands

**Keywords:** plasma membrane, tetraspanin, protein conformation, protein–protein interaction, lymphocyte, super-resolution microscopy

## Abstract

Tetraspanins, including CD53 and CD81, are four-transmembrane proteins that affect the membrane organization to regulate cellular processes including migration, proliferation, and signaling. However, it is unclear how the organizing function of tetraspanins is regulated at the molecular level. Here, we investigated whether recently proposed “open” and “closed” conformations of tetraspanins regulate the nanoscale organization of the plasma membrane of B cells. We generated conformational mutants of CD53 (F44E) and CD81 (4A, E219Q) that represent the “closed” and “open” conformation, respectively. Surface expression of these CD53 and CD81 mutants was comparable to that of WT protein. Localization of mutant tetraspanins into nanodomains was visualized by super-resolution direct stochastic optical reconstruction microscopy. Whereas the size of these nanodomains was unaffected by conformation, the clustered fraction of “closed” CD53 was higher and of “open” CD81 lower than respective WT protein. In addition, KO cells lacking CD53 showed an increased likelihood of clustering of its partner CD45. Interestingly, “closed” CD53 interacted more with CD45 than WT CD53. Absence of CD81 lowered the cluster size of its partner CD19 and “closed” CD81 interacted less with CD19 than WT CD81, but “open” CD81 did not affect CD19 interaction. However, none of the tetraspanin conformations made significant impact on the nanoscale organization of their partners CD19 or CD45. Taken together, conformational mutations of CD53 and CD81 differentially affect their nanoscale organization, but not the organization of their partner proteins. This study improves the molecular insight into cell surface nanoscale organization by tetraspanins.

The plasma membrane is the origin of fundamental cellular processes like ligand-receptor binding, endocytosis, and signaling. These processes depend on proper nanoscale membrane organization that is mediated by cortical actin, lipids, galectins, and tetraspanins (1). The superfamily of tetraspanin proteins interacts in *cis* (on the same cell) with ’partner proteins’ and affect their surface expression, nanoscale organization, and dynamics (2, 3, 4), thereby modulating cell migration, signaling, and immunity (5, 6, 7, 8). Tetraspanins are four-transmembrane proteins that contain two short intracellular tails, two extracellular domains, a small loop (EC1) and a large loop (EC2) with a conserved CCG motif (9, 10). The EC2 has been found to be the predominant site for tetraspanin–protein interactions (11, 12). Previous studies found 80 to 120 nm sized clusters of tetraspanins and partner proteins, called “tetraspanin nanodomains” that form the so-called “tetraspanin web” (13, 14, 15, 16). Although tetraspanin function in cell biology has been widely acknowledged, a major question is how the membrane organizing properties of tetraspanins are regulated at the molecular level?

The first complete 3D structure of a tetraspanin was reported for CD81, a prototypic member of the tetraspanin superfamily important for B cell function (17). CD81 interacts directly with CD19 as part of the B cell coreceptor complex, and CD81 is required for CD19 surface expression and B cell receptor signaling (3). The CD81 structure contains an intramembrane cholesterol binding pocket that was modeled to render CD81 into two conformations (18). When cholesterol is bound, the EC2 “collapses” on the membrane, rendering CD81 “closed.” Without cholesterol the EC2 extends above the membrane, being “open” for interaction. Mutating a single residue, E291Q, abolishes cholesterol binding and skews CD81 into an “open” conformation. Reinforcing the idea of conformation being important for interaction with partner proteins, CD81 was reported in its “open” conformation in a cryo-EM structure together with CD19 (19). Also, the “open” conformation of CD81 was found to increase CD19 surface expression in HEK293 cells (18) although not in Huh-7 cells (20).

Tetraspanin CD53 is expressed on immune cells and interacts with CD45, an important phosphatase in lymphocytes (2). The structure of CD53 also indicated two conformations, however based on a different mechanism (21). Here, the EC2 of CD53 was supported in the “open” conformation by the EC1, irrespective of lipid binding to the binding pocket. A single point mutation of the EC1 (F44E) resulted in loss of EC2 support and therefore collapsed CD53 into a “closed” conformation, which was predicted to lead to reduced CD53–CD2 interaction based on modeling. Similar mutations in the EC1 of CD81 (residues L44A/L45A/Y46A/L47A; named 4A) were postulated to form a “closed” version of CD81, which impaired CD19 maturation (21).

A major question is whether the described “open” and “closed” tetraspanin conformations affect the nanoscale organization of the plasma membrane. Here, we report that conformation of CD53 and CD81 affects their propensity to form tetraspanin nanodomains, but not the size of these domains. In addition, the conformational mutants were found to affect tetraspanin–partner interactions, although the nanoscale organization of the partners (CD19 and CD45) was not altered. This study shows that tetraspanin conformation affects their nanoscale organization which differs between individual tetraspanins.

## Results

### Surface expression of CD53 and CD81 is not dependent on their conformation

**Figure 1:**
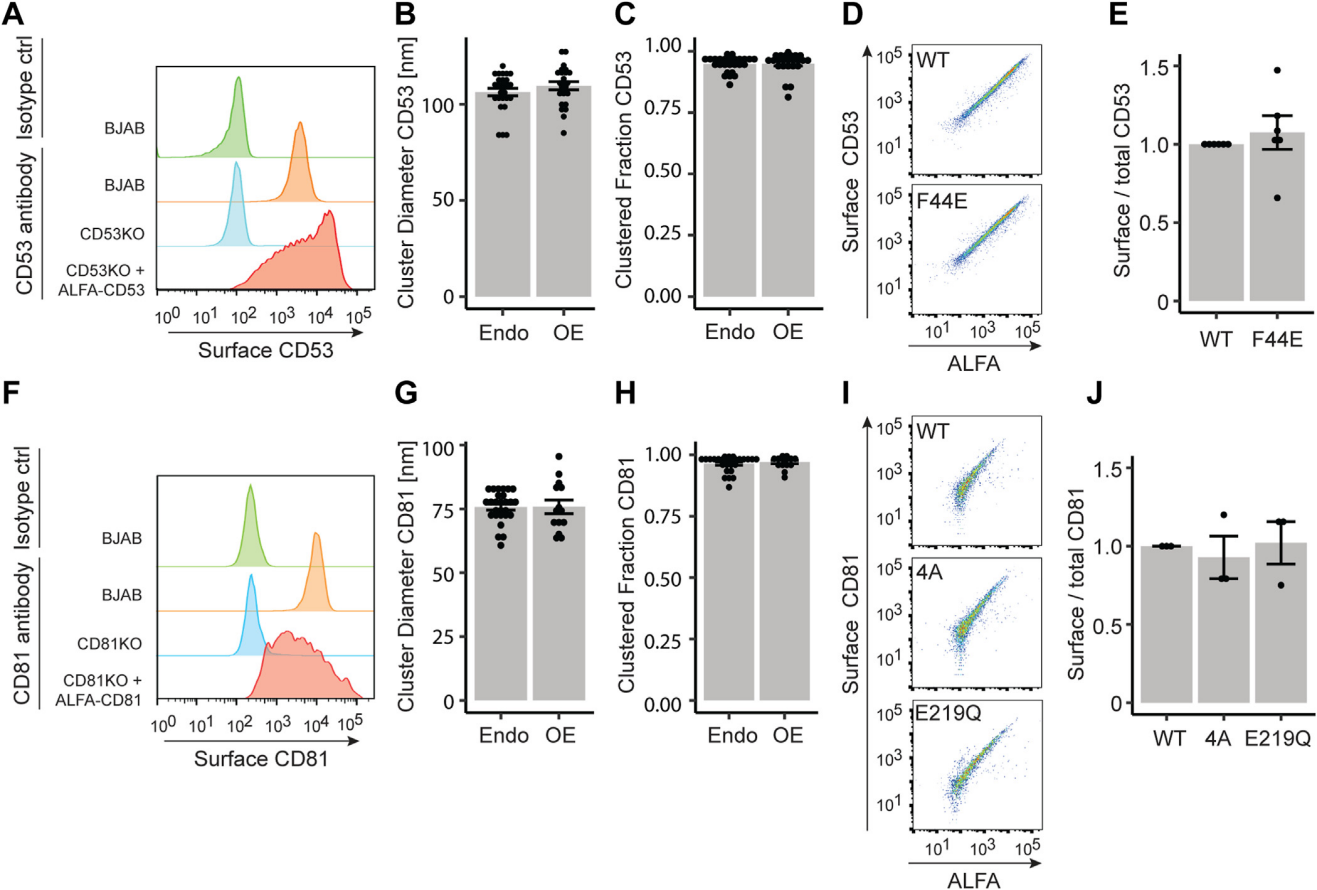
**Surface expression of CD53 and CD81 is not affected by their conformation.***A*, flow cytometry of endogenous surface expression of CD53 in BJAB, CD53KO, and KO cells expressing ALFA-CD53. direct stochastic optical reconstruction microscopy quantification of cluster diameter (B) and fraction of clustered proteins (C) of endogenous and overexpressed CD53 (N = 3, n > 25). *D*, flow cytometry showing the correlation between surface (extracellular antibody, nonpermeabilized) and total (ALFA-staining, permeabilized) CD53 in KO cells transfected with WT or “closed” (F44E) ALFA-CD53. *E*, quantification of (D). Surface to total signal ratio normalized to WT (N = 6). Surface expression (F), cluster diameter (G), and fraction of clustered proteins (H) of endogenous CD81 and ALFA-CD81 transfected into KO cells, analyzed as in (A), (B), and (C) (N = 3, n > 15). *I*, flow cytometry showing the correlation between surface CD81 and total CD81 of KO cells transfected with WT, “closed” (4A), or “open” (E219Q) ALFA-CD81. *J*, quantification of (*I*) as in (*E*) (N = 3).

To study whether tetraspanin conformation affects their behavior, we reintroduced WT and conformational mutants of CD53 and CD81 in KO cells. Since both tetraspanins play an important role in B cell biology, the B cell line BJAB was chosen as model system. KO cell lines were created and surface expression of CD53 and CD81 was reintroduced by transient transfection of ALFA-tagged proteins (Fig. 1, *A* and *F*).

To verify that overexpression of ALFA-tagged tetraspanins in KO cells represents a good model for the endogenous situation, we compared tetraspanin nano-organization using super-resolution dSTORM microscopy of the basal membrane. Cluster size of endogenous CD53 was determined to be ∼110 nm and of endogenous CD81 ∼75 nm, which is in line with former studies (14, 22). The fraction of proteins detected in a cluster was about 0.93 for CD53 and 0.96 for CD81. KO cells overexpressing CD53 or CD81 showed a tetraspanin density (expression) comparable to the endogenous situation (Fig. S1, *A*, *B*, *E*, *F*). No significant differences in cluster diameter or clustered fraction were observed for either CD53 (Fig. 1, *B* and *C*, S1, *C* and *D*) or CD81 (Fig. 1, *G* and *H*, S1, *G* and *H*) between endogenous protein and overexpression, thereby validating our model system.

To investigate whether tetraspanin conformation acts on protein trafficking, we transfected CD53KO cells with WT CD53 and the “closed” (F44E) mutant and quantified surface CD53 signal *versus* total (ALFA-tag) expressed CD53 (Fig. 1*D*). Similarly, WT CD81 and mutants 4A (“closed”) and E219Q (“open”) were introduced in CD81KO cells (Fig. 1*I*). No significant differences between WT and mutants were detected for either CD53 or CD81, showing that their conformation does not affect trafficking to the cell surface (Fig. 1, *E* and *J*).

### Conformational mutations in CD53 and CD81 affect their propensity for nanoscale clustering

**Figure 2:**
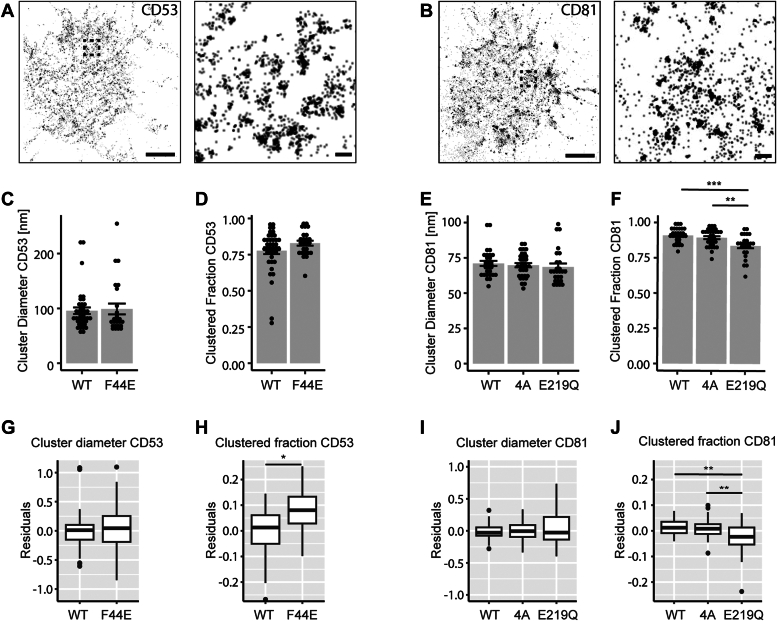
**Conformational mutations in CD53 and CD81 affect their propensity for nanoscale clustering.** direct stochastic optical reconstruction microscopy images showing ALFA-CD53 (A) or ALFA-CD81 (B) in the basal membrane of their respective KO cells. ROI’s in the *left panels* are shown and expanded in the *right panels*. Scale bars represent 2 μm (*left*) and 100 nm (*right*). Quantification of cluster diameter (C) and fraction of clustered proteins of WT and F44E ALFA-CD53 (D) (nonpaired *t* test, N = 3, n > 30). Cluster diameter (E) and fraction of clustered proteins (F) calculated for WT, 4A, and E219Q ALFA-CD81 (ANOVA, N = 3, n > 30). Linear regressions for expression differences and residual analysis were performed (G, H, I, and J) (nonpaired *t* test in G and H, and ANOVA in I and J). ∗*p* ≤ 0.05, ∗∗*p* ≤ 0.01, and ∗∗∗*p* ≤ 0.001.

Since the conformational mutations are predicted to affect the orientation of the EC2, we investigated whether these mutations would affect the nanoscale organization of CD53 and CD81. dSTORM images of ALFA-tagged CD53 and CD81 show clear nanoscale clusters as well as nonclustered proteins on the basal membrane (Fig. 2, *A* and *B*). Surprisingly, no significant difference was observed between the cluster diameter of WT and F44E CD53, both being ∼100 nm (Fig. 2, *C* and *G*). These findings were confirmed using an alternative quantification method (pair correlation analysis) (Fig. S1, *I* and *J*). Whereas cluster size was not affected by conformation, the clustered fraction of mutant F44E seemed higher than WT (Fig. 2*D*). To make sure this subtle difference was not caused by differences in protein expression, the effect of expression was negated by a linear regression on the whole dataset (cluster characteristic *versus* protein expression) and genotype-based residual analysis (exemplified in Fig. S1*B*). A significant mutation-induced increase in clustered fraction was observed for F44E when the effect of expression was accounted for (Fig. 2*H*). Thus, the “closed” conformation does not affect CD53 cluster size but does increase its propensity for nanoscale clustering.

Next, the CD81 conformational mutants were studied and no effect on cluster diameter was found, in line with CD53 (Fig. 2, *E* and *I*). Interestingly, a significantly lower number of clustered CD81 was observed for the “open” mutant (E219Q), whereas the “closed’ mutant” (4A) did not have an effect (Fig. 2, *F* and *J*). Together these data show that CD53 and CD81 conformation does not affect cluster diameter, but affects the propensity for nanoscale clustering.

### CD53 and CD81 differentially affect nanoscale organization of their partner proteins CD45 and CD19

**Figure 3:**
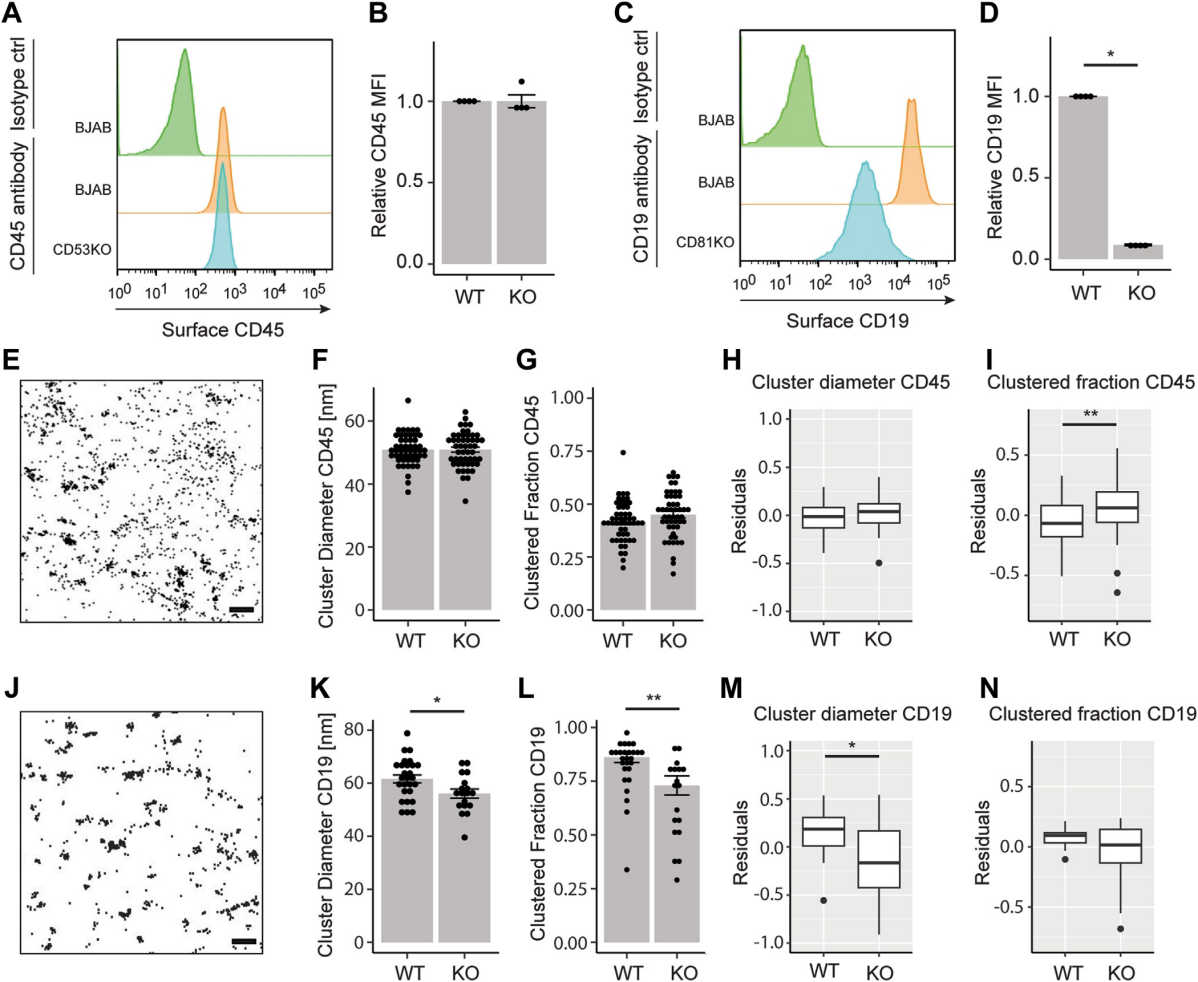
**CD53 and CD81 affect nanoscale organization of their partner proteins CD45 and CD19.***A*, flow cytometry of surface CD45 in BJAB and CD53KO cells. *B*, quantification of (A). Signal normalized to BJAB cells (N = 4). *C*, flow cytometry of surface CD19 in BJAB and CD81KO cells. *D*, quantification of (C) as in (B) (N = 4). *E*, zoomed-in direct stochastic optical reconstruction microscopy images of CD45 on the basal membrane of BJAB cells. The scale bar represents 100 nm. Quantification of cluster diameter of CD45 in BJAB and CD53KO cells (F) and clustered fraction (G) (N = 3, n > 45). Linear regressions for expression differences and residual analysis were performed for cluster diameter (H) and clustered fraction (I). *J*, zoomed-in direct stochastic optical reconstruction microscopy images of CD19 on the basal membrane of BJAB cells. The scale bar represents 100 nm. Cluster diameter (K) and clustered fraction (L) was calculated for endogenous CD19 in BJAB and CD81 KO cells as described in (F) and (G). (N = 3, n > 20). Linear regression and residual analysis for CD19 can be found in (M) and (N). Statistical analysis by nonpaired *t* test. ∗*p* ≤ 0.05 and ∗∗*p* ≤ 0.01.

Next, the membrane organization of partner proteins of CD53 and CD81 was studied. CD45 surface expression was not affected by CD53 knockout (Fig. 3, *A* and *B*). Nanoscale organization of CD45 (Fig. 3*E*) was different from that of CD53 (Fig. 2*A*). The cluster diameter of CD45 was ∼50 nm, lower than that of CD53, and knocking out CD53 did not affect CD45 cluster diameter (Fig. 3, *F* and *H*). In line with this, the clustered fraction of CD45 was lower than that of CD53 (Fig. 2*D*). Interestingly, the fraction of clustered CD45 was significantly higher in the absence of CD53 after linear regression and residual analysis correcting for expression differences (Fig. 3, *G* and *I*). Thus, CD53 does not affect CD45 cluster size, but reduces the formation of CD45 clusters.

Next, the well-established CD81 interaction with CD19 was studied (23, 24). CD81KO cells expressed considerably less surface CD19 (Fig. 3, *C* and *D*), in line with previous studies (3, 25). This significantly lower expression level in CD81KO cells must be considered when analyzing the nanoscale organization of CD19 (Fig. 3, *K* and *L*). When correcting for CD19 expression, CD19 cluster diameter was significantly lower in CD81KO cells (Fig. 3*M*), in contrast to clustered fraction (Fig. 3*L*). Thus, the presence of CD81 increases CD19 cluster size at the cell surface of B cells. Together these data demonstrate that CD53 and CD81 differentially affect the nanoscale organization of their respective partner proteins.

### “Closed” conformations of CD53 and CD81 differentially affect interactions with CD45 and CD19, but not their nanoscale organization

**Figure 4:**
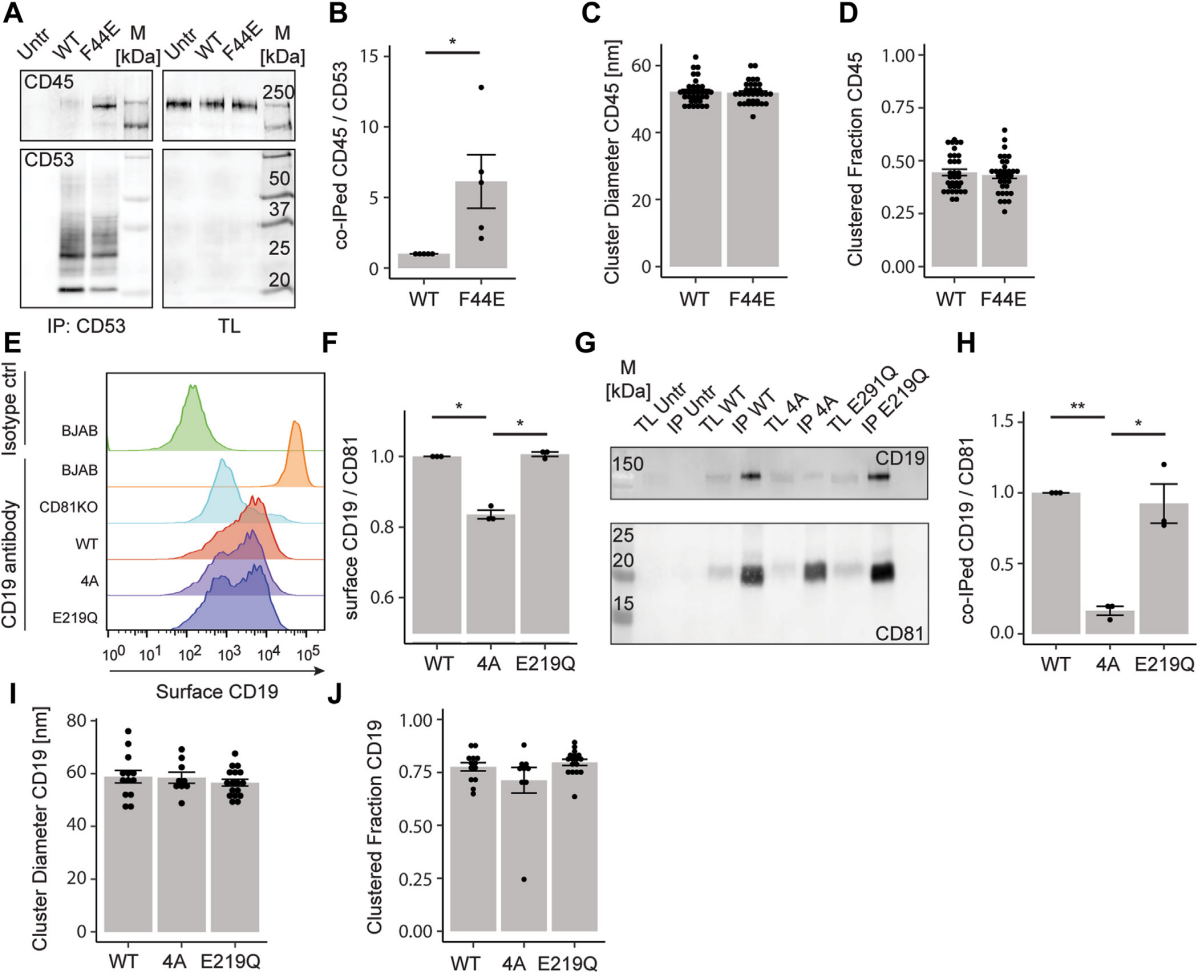
**“Closed” CD53 and CD81 affects interaction with CD45 and CD19 but not their nanoscale organization.***A*, immunoprecipitation (IP) of WT and F44E ALFA-CD53 and co-IP of CD45. *B*, signal of coimmunoprecipitated CD45 divided by signal of immunoprecipitated CD53 normalized to WT (nonpaired *t* test, N = 5). Cluster diameter (C) and fraction of clustered CD45 (D) in CD53KO cells expressing WT and F44E ALFA-CD53 (nonpaired *t* test, N = 3, n > 40). *E*, flow cytometry of CD19 surface expression in BJAB, CD81KO, and KO cells transfected with WT, 4A, and E219Q ALFA-CD81. *F*, quantification of (E). Signal normalized to BJAB cells (ANOVA, N = 3). *G*, IP of WT, 4A, and E219Q ALFA-CD81 and co-IP of CD19. *H*, signal of coimmunoprecipitated CD19 divided by immunoprecipitated CD81 normalized to WT (ANOVA, N = 3). Quantification of cluster diameter (I) and clustered fraction (J) of CD19 in CD81KO cells expressing WT, 4A, and E219Q ALFA-CD81 (ANOVA, N = 3, n > 10). ∗*p* ≤ 0.05 and ∗∗*p* ≤ 0.01. Co-IP, coimmunoprecipitation; TL, total lysate; Untr, untransfected.

Since KO of CD53 and CD81 affected the nanoscale organization of CD45 and CD19, respectively, the effect of tetraspanin conformation on the interaction with, and organization of, their partners was examined. Surprisingly, coimmunoprecipitation experiments showed “closed” CD53 to interact better with CD45 than WT CD53 (Fig. 4, *A* and *B*). This was further substantiated by an enhanced proximity of CD45 to “closed” CD53 as compared to WT CD53 in proximity ligation assays (Fig. S2, *A*, *B* and *C*), although an influence of protein expression differences cannot be excluded (Fig. S3*D*). Despite the clear difference in interaction no differences in CD45 cluster diameter or clustered fraction were observed (Fig. 4, *C* and *D*, S3, *E* and *F*). Next, the effect of CD81 conformation on CD19 was studied. Reintroducing CD81 into CD81KO cells partially rescued CD19 surface expression, in line with previous reports (3). “Closed” CD81 mutant 4A rescued cell surface CD19 to a significantly lower level than the “open” CD81 mutant E219Q (Fig. 4, *E* and *F*). These findings suggest conformation-dependent differences in CD81–CD19 interaction. Indeed, mutant 4A interacted substantially less with CD19 than WT CD81 or mutant E219Q (Fig. 4, *G* and *H*). Supporting these results, we found reduced interaction of CD19 with mutant 4A compared to WT CD81 and E219Q CD81 using proximity ligation assay (Fig. S3, *A*–*C*), although we cannot exclude influences of surface expression differences (Fig. S3*D*). In addition, CD81 conformational mutations had no effect on nanoscale organization of CD19 (Fig. 4, *I* and *J*, S3, *G* and *H*), in line with CD53-CD45. All in all, the conformation of CD53 and CD81 differentially affects their interactions with partner proteins, but not the nanoscale organization of their partners.

## Discussion

Nanoscale organization by tetraspanins affects many important processes at the plasma membrane, including protein stability, clustering, and dynamics. However, it remains unclear how this organization is regulated at the molecular level. Recent studies suggest that the conformation of tetraspanins influences their protein interactions (19, 20, 21, 26), and a major question is whether this dictates nanoscale organization of the plasma membrane. Here, we demonstrate that conformational mutants of CD53 and CD81 differentially affect the probability of these proteins to form nanodomains. Whereas “closed” CD53 showed an increased clustered fraction, the opposite was found for “open” CD81, indicating that conformation affects nanoscale organization in a tetraspanin-specific manner.

The nanoscale organization of partner proteins CD45 and CD19 was affected by knockout of CD53 and CD81, respectively, consistent with the concept of tetraspanins organizing their partner proteins. However, although conformational mutations of CD53 and CD81 clearly affected their interactions with CD45 and CD19, their nanoscale organization was not altered. Our finding that “closed” CD81 interacted far less with CD19 than WT CD81 is in line with Zimmermann *et al.*, and the first time this interaction difference is validated in B cells. CD53 was predicted to interact less with CD2 in a “closed” conformation, whereas we found more interaction of this mutant with CD45 than WT CD53 (21). It is therefore likely that conformation differentially affects tetraspanin–partner interactions.

There can be different explanations for the observation that tetraspanin conformation affects partner protein interactions, but not nanoscale organization. First, CD53 and CD81 may be redundant for the organization of their partner proteins. For example, CD19 has been proposed to also bind to tetraspanins CD9 and CD82 (27). Second, it is possible that these tetraspanins interact with the nonclustered pool of their partner proteins. The latter may explain the bigger pool of clustered CD45 observed in CD53KO cells. Both explanations relate to another open question about the molecular makeup of the tetraspanin web. While one report suggests that tetraspanin clusters are separate domains to those of their partners in proximity (14), other studies imply mixed tetraspanin-partner domains (16, 28, 29). Our data favors the first explanation for CD53-CD45 and CD81-CD19, with cluster sizes differing between tetraspanins and their respective partners. Multichannel super-resolution microscopy techniques like dual-color dSTORM or DNA paint are expected to make an important contribution to resolve this further.

Our data indicate that the described “open” and “closed” conformations do not necessarily correspond to “on” or “off” switches for interaction. This concept is supported by two other reported structures of tetraspanins CD9 and Tspan15. The structure of CD9 is homologous to CD81 and was crystalized in a “closed” conformation (30), however in complex with partner protein EWI-F, it adopts a semi-open conformation (29). Conversely, the structure of Tspan15 was resolved in a “closed” conformation in complex with its partner ADAM10 (26). This, together with the structure for CD81 in complex with CD19, indicates that both the “open” and the “closed” conformation can be the interactive variant. Future structural studies on tetraspanin–partner complexes as well as proposed conformational mutants will be essential to further substantiate this hypothesis.

Conformation-controlled interactions support the exciting idea of regulating nanoscale organization of the plasma membrane by tetraspanin conformation. Several mechanisms have been suggested to affect tetraspanin conformation. Three out of four tetraspanin structures reported to date found hydrophobic binding pockets in the intramembrane space suitable for binding lipids like cholesterol (18, 21, 26, 30, 31). The dynamic equilibrium between bound and nonbound lipids could determine the conformational dynamics of tetraspanins and therefore their interactions (32). Recently suggested is the involvement of a conserved small intracellular loop, which may affect conformation by electrostatic interactions (33). A third mechanism is that of lipid surroundings and membrane curvature (34, 35, 36, 37). As tetraspanins readily adopt a so-called ice-cone shape, it has been proposed that they either induce or follow preexisting membrane curvature, aided by distinct lipids like gangliosides (34). Further adding to the association of tetraspanins with certain lipids is the fact that many of them are palmitoylated at membrane-proximal cysteines (38).

While we report tetraspanin conformation to affect their membrane organization by nanoscale clustering, there are also limitations to our study. First, misinterpretation of protein density fluctuations as nanoclusters has been reported in dSTORM microscopy analysis (39). However, similar-sized nanoclusters have been described before in B cells, HepG2 and HaCaT cells for CD19, CD81, and CD53 by stimulated emission depletion microscopy, which is way less susceptible to this limitation (14, 16, 40). For CD45, we validated that cluster formation was largely unaffected by lowering the antibody concentrations by 100-fold (Fig. S4), in line with nonrandom protein distribution. Second, the “closed” and “open” tetraspanin mutants have not been resolved yet in cryo-EM structures. An important next step in this exciting research direction is to couple the observed differences in interactions and membrane organization to cell function.

Taken together, our findings demonstrate that conformation of CD53 and CD81 affects their nanoscale organization. Clusters formed by conformational mutants are not different in size but in the probability to form clusters. Interactions with CD45 and CD19, respectively, are differentially affected by “closed” mutants; however, these differences in interaction do not influence the nanoscale organization of those partner proteins. Thus, conformation of CD53 and CD81 affects their nanodomain formation and interaction with its partners but does not affect the nanoscale organization of CD45 and CD19.

## Experimental procedures

Detailed experimental procedures are provided in the Supporting Information.

### Cell culture and transfection

BJAB cells (DSMZ, cat: ACC757, mycoplasma-negative) were cultured at 37 °C and 5% CO_2_ in RPMI-1640 + 10% fetal bovine serum + 1% antibiotics/antimycotics (AA) + 1% ultraglutamine. Transfection of 5 × 10^6^ cells with 2 μg plasmid was performed with the SF Cell Line 4D-Nucleofector X Kit L (Lonza) and the AMAXA Nucleofector biosystem (Program DS104). Cells were subsequently cultured for 16 to 24 h in growth medium without AA. CD81 and CD53 KO BJAB cells were generated by CRISPR/Cas9 technology as described before (41, 42) using guide RNA’s listed in Table S1 and verified by flow cytometry.

### dSTORM microscopy

0.5 × 10^6^ cells were adhered to poly-L-lysine–coated cover slides. Extracellular epitopes were stained in suspension on ice as described for flow cytometry and then adhered and fixed on ice for 1 h in 0.1% glutaraldehyde and 4% paraformaldehyde in 0.2 M phosphate buffer pH 7.4. For intracellular epitopes, cells were first adhered at 37 °C and then fixed as described above. Cells were washed with PBS and quenched by a 30-min incubation with 100 mM glycine, 100 mM NH_4_Cl, and 0.1% Triton X-100 in PBS. Samples were blocked and stained in PBS + 50 mM glycine + 3% bovine serum albumin + 2% Human Serum (HS). Intracellular stainings were supplemented with 0.1% Triton X-100. Samples were stored in PBS + 0.1% paraformaldehyde at 4 °C. Before imaging, samples were washed and quenched. Coverslips were mounted in a magnetic sample holder and imaged in 1 ml OxEA buffer (43). dSTORM microscopy was performed as described in (38). To make sure samples with overexpressed tetraspanins have a similar expression range as the endogenous situation, the brightest and dimmest cells were not imaged. Localization data was extracted using the ThunderSTORM module in FIJI (44) (https://imagej.net/software/fiji/). Images were reconstructed using the averaged shifted histograms method with a rendering pixel size of 10 nm. Cluster characteristics were calculated by the DBSCAN function (ϵ = 50 nm, minpts = 5) in the RSMLM-package in R (45). Linear regressions were performed on the pooled data of cluster diameter and clustered fraction *versus* localization density. Genotype-specific effects were visualized by residual analysis of the regression model. Pair correlation analysis was performed with the SpatStat package in R.

### Statistics

Bars and error bars represent the mean and SEM. Statistical testing was performed on non-normalized data and is indicated in the figure legends. ∗*p* ≤ 0.05, ∗∗*p* ≤ 0.01, and ∗∗∗*p* ≤ 0.001. N refers to the number of independent experiments and n to the number of analyzed cells per genotype.

## Data availability

All data are contained within the article.

## Supporting information

This article contains supporting information (41).

## Conflict of interest

The authors declare that they have no conflicts of interest with the contents of this article.
