# Supplementary material for: The conformation of tetraspanins CD53 and CD81 differentially affects their nanoscale organization and interaction with their partners
Source: J Biol Chem. 2024 Aug 17;300(9):107685. doi: 10.1016/j.jbc.2024.107685 (PMC11416636; doi:10.1016/j.jbc.2024.107685)
Supplement: Supporting Figures [file mmc1.docx]

The conformation of tetraspanins CD53 and CD81 differentially affects their nanoscale organization and interaction with their partners

Fabian Schwerdtfeger^1^, Ilse A. Hoogvliet^1^, Sjoerd van Deventer^1,+^, Annemiek B. van Spriel^1,+,^*

**Supporting information**

Supporting information figures 1, 2, 3 and 4, supplemental table 1, extended and supplemental experimental procedures


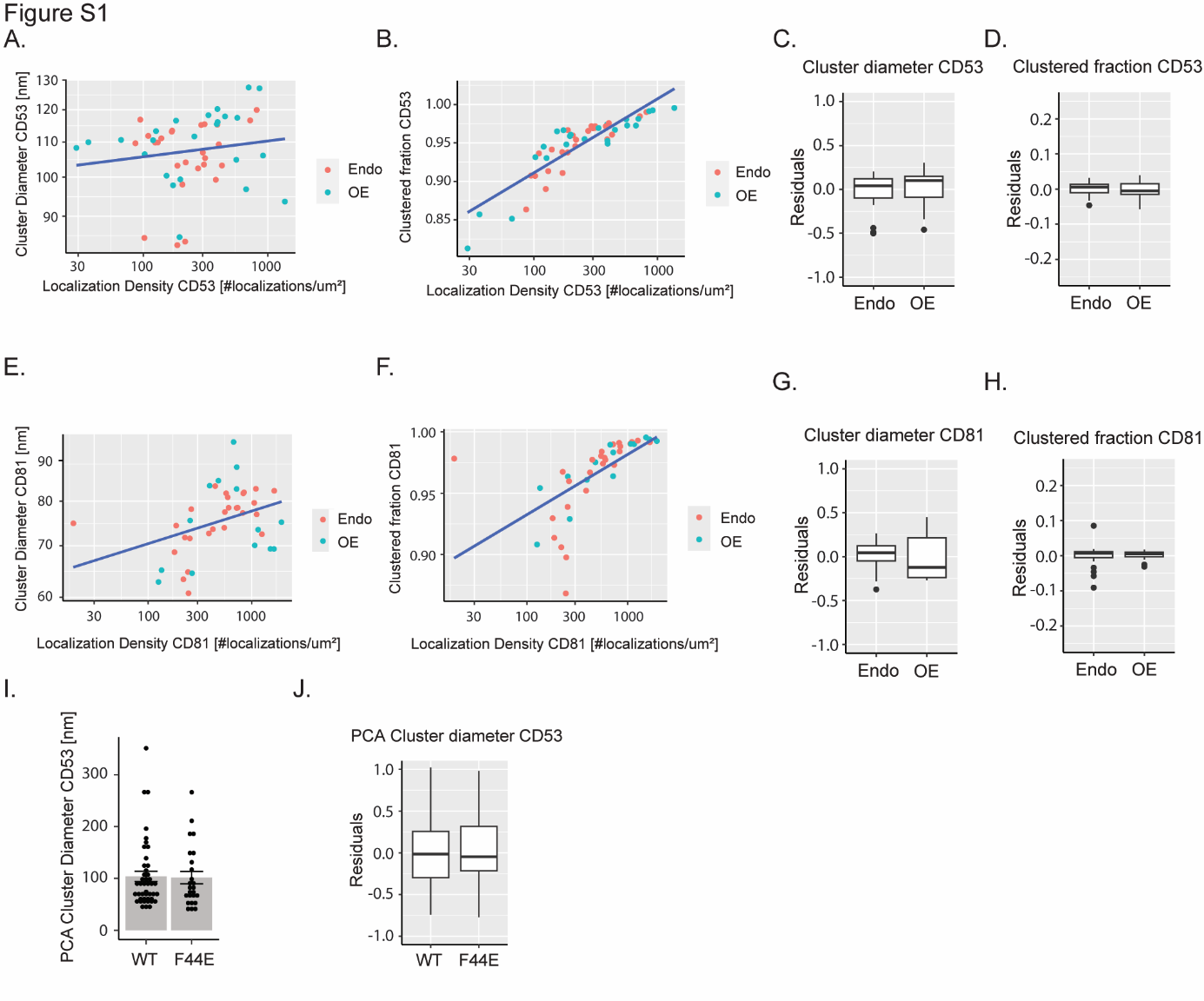


**Figure S1:** Supporting information for the analysis of tetraspanin nanoscale organization.

Scatterplots showing the cluster diameter (**A**) and clustered fraction (**B**) versus localization density of CD53 in the basal membrane of BJAB cells (Endogenous, red) or CD53KO BJAB cells overexpressing ALFA-CD53 (OE, blue). A linear regression based on all data points is plotted. (**C**) Residuals of the regression model for the endogenous and overexpressed samples are depicted for the cluster diameter and clustered fraction (**D**) (N=3, n>25). Plots and residuals for CD81 (**E-H**), (N=3, n>15). Cluster diameter of CD53 (**I**) and residuals (**J**) was analyzed by paired correlation analysis (PCA).


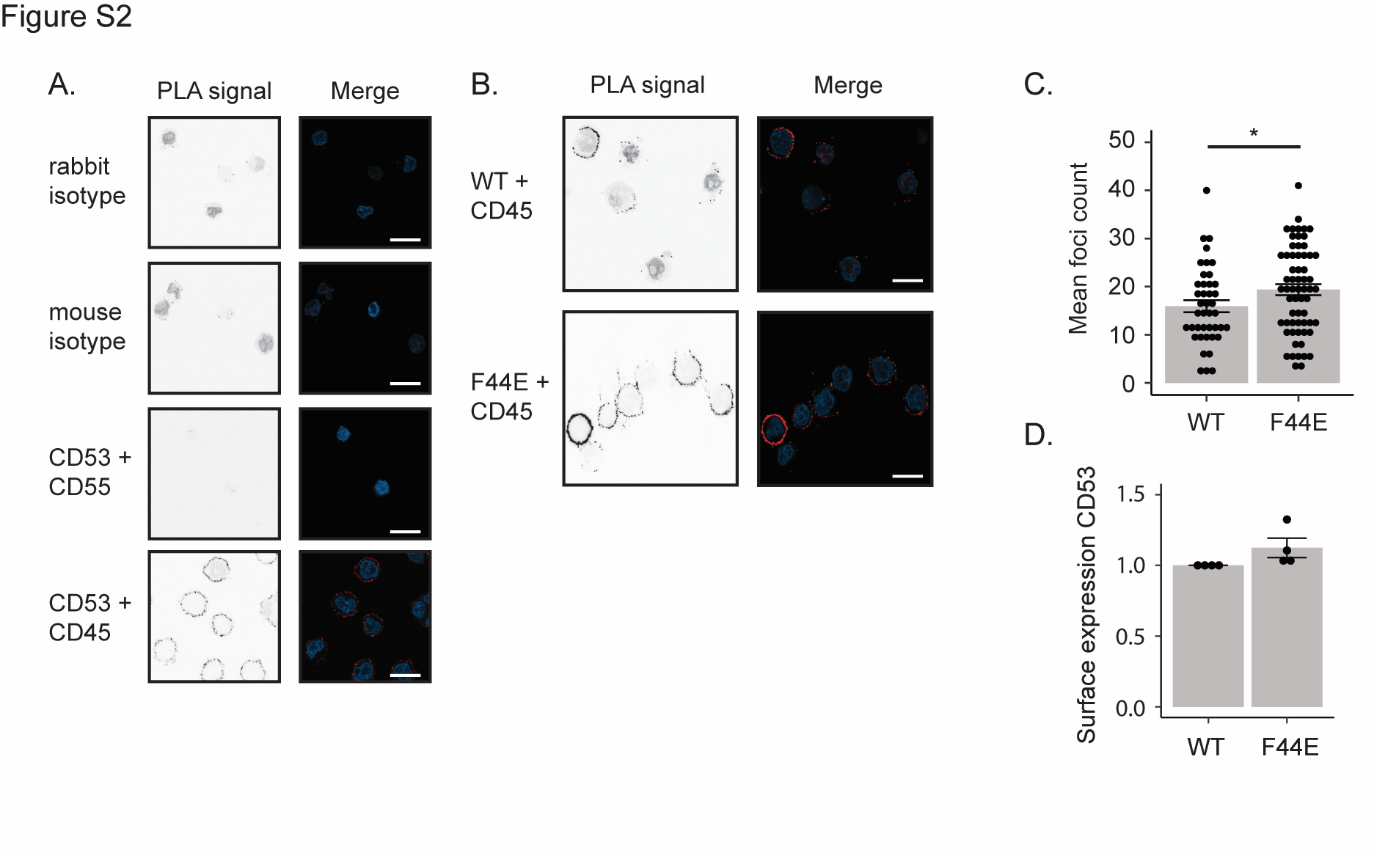


**Figure S2:** Supporting information for the analysis of tetraspanin-partner CD53-CD45 interaction.

(**A**) Confocal microscopy of Proximity Ligation Assays (PLA) on BJAB cells. PLA signal (left) and merged (DAPI in blue, PLA signal in red, right) of isotype controls, negative control (CD53-CD55, a GPI-anchored protein) and sample of interest (CD53-CD45). Scale bar= 5μm. (**B**) Confocal microscopy of PLA (left column) assessing the proximity of overexpressed WT and ‘closed’ CD53 (F44E) to endogenous CD45. Scale bar= 5μm. (**C**) Quantification of the number of PLA foci per transfected cell (Student’s t-test, N=4, n> 40). (**D**) Flow cytometry analysis showing the relative expression levels of surface CD53 in the samples in (B) (N=4). Bar plots show mean ± sem. * P≤0.05.


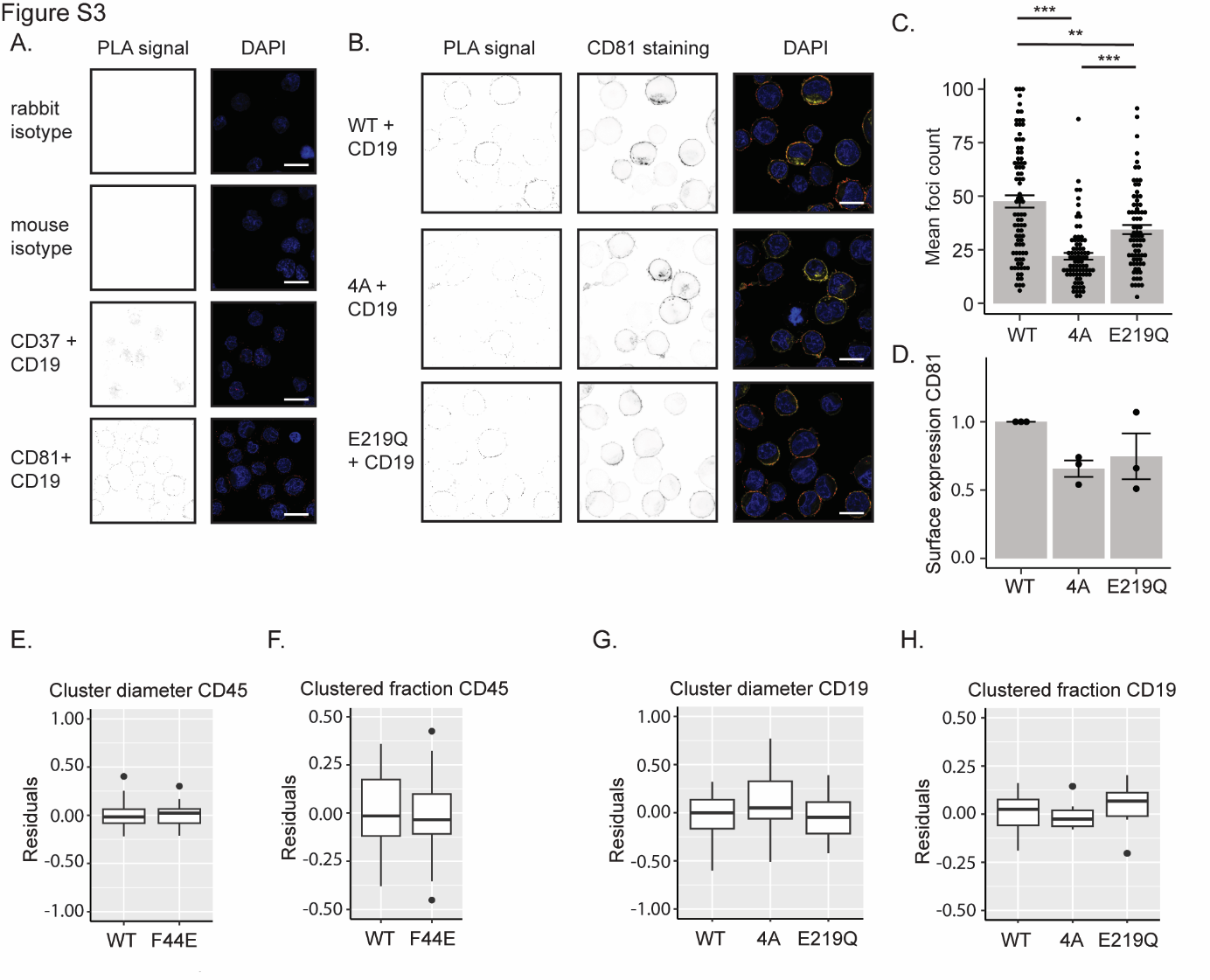


**Figure S3:** Supporting information for the analysis of tetraspanin-partner interactions and organization.

(**A**) Confocal microscopy of Proximity Ligation Assays (PLA) on untransfected BJAB cells. PLA signal (left) and merged (DAPI in blue, right) of isotype controls, negative control (CD81-CD37, another highly expressed tetraspanin) and sample of interest (CD81-CD19). Scale bar= 5μm. (**B**) Confocal microscopy of PLA (left column) assessing the proximity of overexpressed WT CD81, ‘closed’ (4A) and ‘open’ (E219Q) ALFA-CD81 (middle column) to endogenous CD19. Scale bar= 5μm. (**C**) Quantification of the number of PLA foci per transfected cell (ANOVA, N=3, n> 75). (**D**) Flow cytometry analysis showing the relative expression levels of surface CD81 in the samples in (B) (N=3). To eliminate the effect of differences in CD45 surface expression from the DBSCAN quantification of its cluster characteristics, linear regressions were performed of the cluster diameter and clustered fraction versus the localization density on the pooled data for all samples. Residuals of the regression model for the different samples separately are depicted for the cluster diameter (**E**) and clustered fraction (**F**) (N=3, n>40). The results of a linear regression and residual analysis for CD19 in the different samples (**G, H**) (N=3, n>10). Bar plots show mean ± sem. ** P≤0.01 and ***P≤0.001.


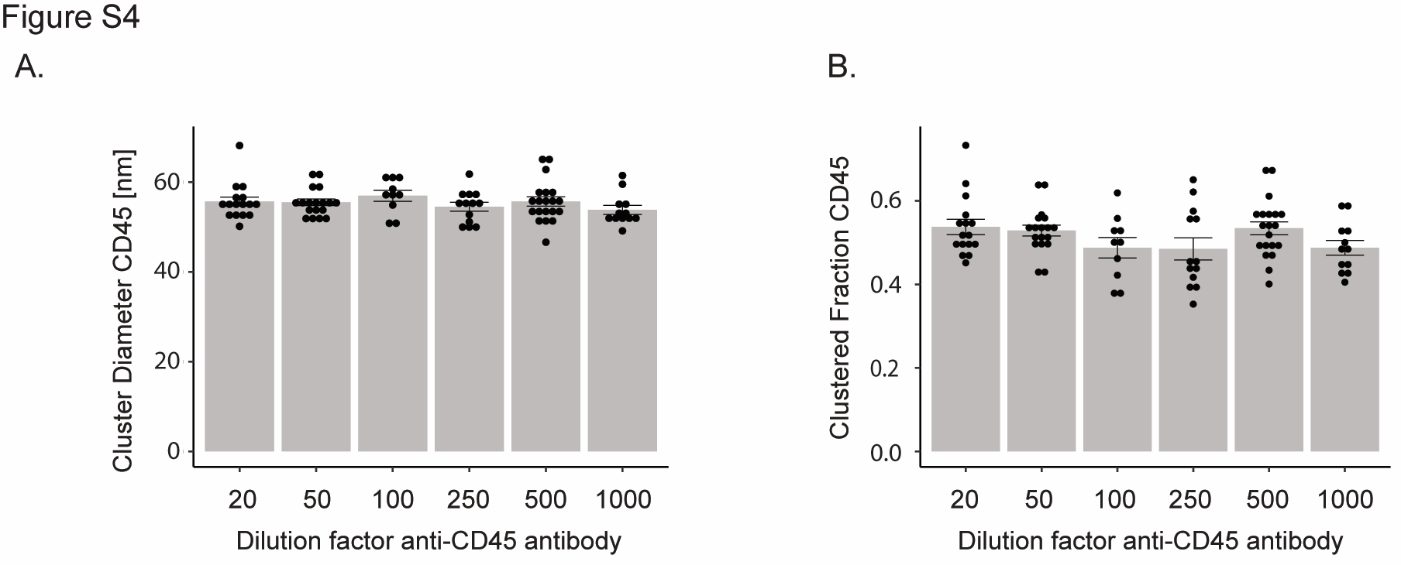


**Figure S4:** Cluster parameters with varying dilutions of anti-CD45 antibody.

(**A**) Cluster diameter of CD45 on the surface of BJAB WT cells with varying anti-CD45 antibody concentrations. (**B**) Clustered fraction of CD45 on the surface of BJAB WT cells with varying anti-CD45 antibody concentrations. Stock concentration is 0.1mg/ml. Bar plots show mean ± sem.

**Supplemental Table S1**

| **Antibody:** | **Source:** | **Identifier:** | **Use:** |
| --- | --- | --- | --- |
| Rabbit anti-human CD19 | abcam | Clone: EPR5906  Cat#: ab134114 | WB: 1:1000 |
| Rabbit anti-human CD19 | ABNOVA | Clone: polyclonal  Cat#: H00000930-D01P | PLA: 10 μg/ml |
| Mouse anti-human CD19 AF647 | Novus Biologicals | Clone: CB19  Cat#: NBP2-25196AF647 | IF/FC: 1:100 |
| Mouse anti-human CD45 AF488 | Novus Biologicals | Clone: F10-89-4  Cat#: NBP1-28509AF488 | IF/FC: 1:50 |
| Rabbit anti-human CD45 | Cell Signaling Technologies | Clone: D9M8I  Cat#: 13917S | WB: 1:1000 |
| Mouse anti-human CD53 | Bio-Rad | Clone: MEM53  Cat#: MCA723G | FC: 10 μg/ml |
| Mouse anti-human CD53 AF647 | Novus Biologicals | Clone: MEM53  Cat#: NB500-393AF647 | IF/FC: 10 μg/ml |
| Mouse anti-human CD81 AF647 | Novus Biologicals | Clone: M38  Cat#: NBP1-44861AF647 | IF/FC: 1:100 |
| Mouse anti-human CD81 | BD Biosciences | Clone: JS-81  Cat#: 555675 | PLA: 10 μg/ml |
| Mouse anti-human CD37 | Homemade | Clone: WR17 | PLA: 10 μg/ml |
| Mouse anti-human CD45 | Abcam | Clone: MEM28  Cat#: ab8216 | PLA: 2 ug/ml |
| Rabbit anti-human CD53 | Novus Biologicals | Clone: 161-2 (53/2) Cat#: NBP3-12077 | PLA: 2μg/ml |
| Mouse anti-human CD55 | Thermo Fisher Scientific | Clone: MEM-118  Cat#: MA1-19127 | PLA: 10μg/ml |
| Anti-ALFA-Atto488 | Nano Tag Biotechnologies | Clone: 1G5  Cat#: N1502-At488-L | IF/FC: 1:250 |
| Anti-ALFA-AZdye568 | Nano Tag Biotechnologies | Clone: 1G5  Cat#: N1502-AF568-L | IF/FC: 1:250 |
| Anti-ALFA-AF647 | Nano Tag Biotechnologies | Clone: 1G5  Cat#: N1502-AF647-L | IF/FC: 1:250  WB: 1:2000 |
| Goat-α-Mouse -IgG1-Alexa488 | Thermo Fisher Scientific | Cat#: A21121 | IF/FC: 1:400 |
| Goat-anti rabbit IRDye800 | Li-Cor | Cat#: 926-32211 | WB: 1:5000 |
|  |  |  |  |
| **Protein** | **Guide RNA sequence** |  |  |
| CD53 | TGATAGAGCCCATGCAGCCCAGG  GTGAGTCCTTACAGCAGATGTGG |  |  |
| CD81 | CACCGTGACAGCGCCCACAGCGATG  AAACCATCGCTGTGGGCGCTGTCAC |  |  |

IF: Immune Fluorescence / FC: Flow Cytometry / WB: Western Blot / PLA: Proximity Ligation Assay

AF: AlexaFluor

**Extended experimental procedures:**

Plasmids and mutagenesis

Expression plasmids containing WT CD81 and CD53 with an N-terminal ALFA-tag in pCDNA3.1(+) were obtained as described before (1). Point mutations were introduced using the Q5 Site-Directed Mutagenesis Kit (New England Biolabs) and custom primers and verified by Sanger sequencing.

Flow cytometry

Samples containing 2x10^5^ cells were blocked for 30min on ice in PBS + 1% BSA + 2% human serum (HS), stained in blocking buffer for 30min on ice (antibodies and dilutions in Table S1) and analyzed on a FACSVerse or FACSLyric (BD Biosciences). Data was analyzed using FlowJo X (Tree Start Inc.). Intracellular staining was performed on cells fixed in 4% paraformaldehyde (PFA) in PBS for 30min on ice and subsequent steps were performed in the presence of 0.5% saponin.

Immunoprecipitation and Western blot

Immunoprecipitation of ALFA-tagged CD81 and CD53 was performed after lysing 5x10^6^ transfected cells in 1 ml 1% Brij97, 10 mM TRIS pH 7.5 and 150 mM NaCl with either 1 mM EDTA (for CD53) or 2 mM CaCl_2_ + 2 mM MgCl_2_ (for CD81) by a 30min incubation on ice with intermittent vortexing. After 5min centrifugation at 6000 rpm, ALFA selector ST beads (NanoTag Biotechnologies) were added and incubated for 2h rotating at 4^o^C. Beads were washed with lysis buffers containing 0.1% Brij97. Proteins were eluted by 10min incubation at 95^o^C in 1x sample buffer + 2.5% β-mercaptoethanol. Samples were run on a 10% (CD53) or 12% (CD81) SDS-PAGE and transferred to a PVDF membrane (GE Healthcare). Proteins were stained with antibodies and buffers (Table S1), visualized on a Typhoon 5 (Amersham) and quantified using FIJI.

Proximity ligation assay

Half a million transfected cells were adhered to poly-L-lysine-coated coverslides and subsequently fixed by incubation with 4% PFA in PBS for 30 minutes. After thorough washing with PBS, Proximity Ligation Assays (PLA) were performed using the Duolink In Situ PLA FarRed kit (Sigma-Aldrich), according to manufacturer’s instructions. In short, cells were blocked for 30 minutes in the provided DuoLink Blocking solution, which was supplemented with 2% human serum. Cells were subsequently stained for 30 min in the provided Antibody Diluent supplemented with 2% human serum and mixtures of two primary antibodies at a concentration of 2 or 10 µg/ml (listed in Table S1). The incubation with DNA-coupled secondary antibodies provided by the kit was also performed in the presence of 2% human serum and was combined with an Alexa568-labeled nanobody against the ALFA-tag to identify transfected cells. After performing the PLA, samples were stained with DAPI, washed, and embedded in mowiol. Cells were imaged on a Zeiss LSM900 confocal microscope using the Airyscan detector (Zeiss) and a 63x oil immersion 1.4NA objective. Data was analyzed using a FIJI-based routine consisting of an intensity threshold to separate PLA foci from background signal, a watershed to separate touching foci and a size threshold on the identified objects to select genuine PLA spots.

1. van Deventer, S., Hoogvliet, I. A., van de Voort, M., Arnold, F., Ter Beest, M., andvan Spriel, A. B. (2023) N-Glycosylation-dependent regulation of immune-specific tetraspanins CD37 and CD53 Biophys J 10.1016/j.bpj.2023.11.3399
